# The Potential Protective Role of RUNX1 in Nonalcoholic Fatty Liver Disease

**DOI:** 10.3390/ijms22105239

**Published:** 2021-05-15

**Authors:** Laia Bertran, Angela Pastor, Marta Portillo-Carrasquer, Jessica Binetti, Carmen Aguilar, Salomé Martínez, Margarita Vives, Fàtima Sabench, José Antonio Porras, David Riesco, Daniel Del Castillo, Cristóbal Richart, Teresa Auguet

**Affiliations:** 1 Grup de Recerca GEMMAIR (AGAUR)—Medicina Aplicada (URV), Departament de Medicina i Cirurgia, Institut d’Investigació Sanitària Pere Virgili (IISPV), Universitat Rovira i Virgili (URV), 43007 Tarragona, Spain; laia.bertran@urv.cat (L.B.); angela.cuellar98@gmail.com (A.P.); marta.portillo.carrasquer@gmail.com (M.P.-C.); caguilar.hj23.ics@gencat.cat (C.A.); crichart.hj23.ics@gencat.cat (C.R.); 2 Servei Medicina Interna, Hospital Universitari Joan XXIII Tarragona, Mallafré Guasch, 4, 43007 Tarragona, Spain; jessica.binetti@gmail.com (J.B.); aporras.hj23.ics@gencat.cat (J.A.P.); david_riesco@hotmail.com (D.R.); 3 Servei Anatomia Patològica, Hospital Universitari Joan XXIII Tarragona, Mallafré Guasch, 4, 43007 Tarragona, Spain; mgonzalez.hj23.ics@gencat.cat; 4 Servei de Cirurgia, Hospital Sant Joan de Reus, Departament de Medicina i Cirurgia, URV, IISPV, Avinguda Doctor Josep Laporte, 2, 43204 Reus, Spain; mvives@gmail.com (M.V.); fatima.sabench@urv.cat (F.S.); danieldel.castillo@urv.cat (D.D.C.)

**Keywords:** RUNX1, NAFLD, liver, jejunum, metabolism, gut–liver axis

## Abstract

The pathogenic mechanisms underlying nonalcoholic fatty liver disease (NAFLD) are beginning to be understood. RUNX1 is involved in angiogenesis, which is crucial in inflammation, but its role in nonalcoholic steatohepatitis (NASH) remains unclear. The aim of this study was to analyze RUNX1 mRNA hepatic and jejunal abundance in women with morbid obesity (MO) and NAFLD. RUNX1, lipid metabolism-related genes, and TLRs in women with MO and normal liver (NL, *n* = 28), NAFLD (*n* = 41) (simple steatosis (SS, *n* = 24), or NASH (*n* = 17)) were analyzed by RT-qPCR. The RUNX1 hepatic expression was higher in SS than in NL or NASH, as likewise confirmed by immunohistochemistry. An increased expression of hepatic FAS was found in NAFLD. Hepatic RUNX1 correlated positively with FAS. There were no significant differences in the jejunum RUNX1 expressions in the different groups. Jejunal FXR expression was lower in NASH than in NL, while the TLR9 expression increased as NAFLD progressed. Jejunal RUNX1 correlated positively with jejunal PPARγ, TLR4, and TLR5. In summary, the hepatic expression of RUNX1 seems to be involved in the first steps of the NAFLD process; however, in NASH, it seems to be downregulated. Our findings provide important insights into the role of RUNX1 in the context of NAFLD/NASH, suggesting a protective role.

## 1. Introduction

Nonalcoholic fatty liver disease (NAFLD) is currently the most prevalent chronic liver disease worldwide, with an estimated global prevalence of approximately 25%, and as high as 95% in individuals with morbid obesity [1]. NAFLD and metabolic syndrome (MetS) are highly related, since obesity and type 2 diabetes, two MetS components, are the main risk factors for NAFLD [2]. NAFLD comprises a wide spectrum of liver pathologies, ranging from simple steatosis (SS), a mild condition identified by the presence of <5% hepatic steatosis without evidence of hepatocellular injury, to nonalcoholic steatohepatitis (NASH), defined as the advanced stage in the chronic NAFLD spectrum. NASH may progress to liver cirrhosis, fibrosis, and ultimately hepatocellular carcinoma (HCC), and it is emerging as an important cause of liver transplants [2,3,4]. Therefore, NAFLD has become a serious public health problem.

The underlying precise mechanisms of NAFLD pathogenesis are beginning to be understood. The current working hypothesis is based on a ‘‘parallel multiple-hit model’’, in which various factors that act in parallel— such as obesity, sedentary lifestyle, high-fat diet, insulin resistance, and hepatic lipid accumulation—lead to the development of hepatic inflammation and fibrosis [5]. The progression to NASH is linked to systemic inflammation and is associated with other pathological processes, such as innate immunity alterations, endoplasmic reticulum stress and mitochondrial dysfunction, or intestinal dysbiosis and toll-like receptor (TLR) activation [6,7,8,9,10]. Since there are currently no FDA-approved therapies for treating NAFLD/NASH, improving our knowledge of the NAFLD pathogenesis is necessary for developing treatment strategies for the prevention of its development.

Runt-related transcription factor 1 (RUNX1), also known as acute myeloid leukemia 1, is a sequence-specific DNA-binding transcription factor that can act as an oncogene or tumor suppressor, depending on its interaction with specific co-regulatory proteins. For example, low levels of RUNX1 have been reported in HCC, indicating its suppressive function [11,12].

RUNX1 is a powerful and pivotal regulator of hematopoiesis and angiogenesis [13,14]. Angiogenesis, or new blood vessel formation, is a crucial aspect of inflammation in remodeling damaged tissue. Changes in hepatic vascular architecture have been linked to the progression of fibrosis, cirrhosis, and HCC [15,16]. It was reported that NASH is associated with the enhanced expression of pro-angiogenic cytokines. However, the precise mechanisms that regulate angiogenesis, inflammation, and fibrogenesis in NASH pathology remain unclear [17,18].

**Figure 1:**
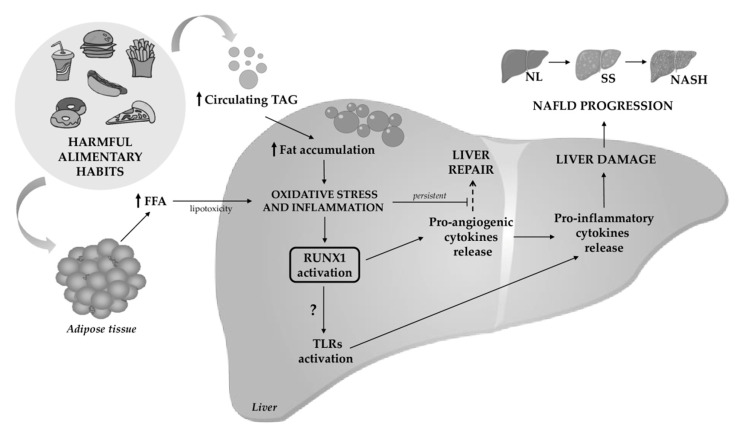
RUNX1 is involved in inflammation-mediated NAFLD. Harmful eating habits lead to high levels of circulating triglycerides (TAG), which accumulate in the liver, causing oxidative stress and inflammation. These two processes activate RUNX1, which turns on the angiogenesis pathway to try to repair injuries. At the same time, there is a growth of the adipose tissue that releases free fatty acids (FFA) that reach the liver, leading to lipotoxicity that promotes inflammation. A persistent inflammation state inhibits the tissue remodeling and promotes the release of pro-inflammatory cytokines, which leads to liver tissue damage and enhances the progression of NAFLD to NASH [13,14,15,16,19]. On the other hand, it was also shown that RUNX1 is a positive regulator of TLR4, signaling for the induction of the inflammatory response [21]. TLR are involved in the NAFLD pathogenesis [22].

Oxidative stress and inflammation-driven pathological angiogenesis play important roles in NAFLD progression [19]. A previous report showed that certain angiogenesis-associated genes are differentially expressed in NASH, such as peroxisome proliferator-activated receptor γ (PPARγ), a well-characterized gene involved in fatty acid uptake and transport [20]; and RUNX1, which has been suggested to be involved in enhanced inflammation and disease severity [16]. Figure 1 hypothesizes the role of RUNX1 in the pathogenesis of NAFLD.

Additionally, it was also shown that RUNX1 is a positive regulator of the TLR4 pathway for the induction of the inflammatory response [21]. The roles of TLRs in NAFLD are well established since there is a clear relation between gut dysbiosis, bacterial-derived component release, TLR activation, and the inflammatory response involved in chronic liver diseases [22].

The liver and intestine communicate in a bidirectional way through the biliary tract, portal vein, and other components of the gut–liver axis. Lipopolysaccharide, which activates TLR4, can be transported by bacterial extracellular vesicles, spherical structures produced by eukaryotic and prokaryotic cells that transfer information to distant cells and may represent novel players in NAFLD development and progression, such as liver inflammation, angiogenesis, and fibrogenesis [23].

Together, these facts suggest that the RUNX1 signaling pathway may be involved in the NAFLD pathogenesis. Therefore, the main aim of this study was to analyze the relative mRNA hepatic abundance of RUNX1 in women with morbid obesity (MO) and NAFLD, with different degrees of liver damage. Additionally, given the important link between the gut and the liver in NAFLD development, we wanted to analyze the relative mRNA jejunal abundance of RUNX1 in this cohort of patients. Finally, we aimed to ascertain the possible relationship of the RUNX1 expression with the main liver lipid metabolism-related genes and the TLR expressions.

## 2. Results

### 2.1. Baseline Characteristics of Subjects

The clinical characteristics and biochemical measurements of the population studied are shown in Table 1. We first classified the subjects according to hepatic histology as MO with normal liver (NL) (*n* = 28) and MO with NAFLD (*n* = 41), which were comparable in terms of weight, body mass index (BMI), glucose, insulin, homeostatic model assessment method 1 of insulin resistance (HOMA1-IR), glycosylated hemoglobin (HbA1c), cholesterol, high density lipoprotein cholesterol (HDL-C), low density lipoprotein cholesterol (LDL-C), triglycerides, aspartate aminotransferase (AST), alanine aminotransferase (ALT), gamma-glutamyltransferase (GGT), alkaline phosphatase (ALP), lactate dehydrogenase (LDH), systolic blood pressure (SBP), diastolic blood pressure (DBP), interleukin (IL) 1β, IL-6, IL-8, IL-10, tumor necrosis factor α (TNF-α), adiponectin, and resistin. However, biochemical analyses indicated that patients with NAFLD had significantly higher levels of fasting glucose (*p* = 0.026), ALP (*p* = 0.009), and IL-8 (*p* = 0.001) than patients with NL histology.

In order to add new knowledge about the role of RUNX1 in hepatic steatosis, we then subclassified our patients with MO and NAFLD into SS (*n* = 24) and NASH (*n* = 17) (Table 1). Fasting glucose (*p* = 0.021), ALT (*p* = 0.042), and ALP (*p* = 0.001) levels were significantly higher in the SS group than in NL subjects. ALP (*p* = 0.008) levels were significantly higher in SS than in NASH. IL-8 (*p* = 0.037) levels were higher in NASH than in SS. IL-8(*p* = 0.001) levels were also significantly higher in NASH than in NL.

### 2.2. Evaluation of the Relative mRNA Abundance of RUNX1 in the Liver and Jejunum According to Hepatic Histology

**Figure 2:**
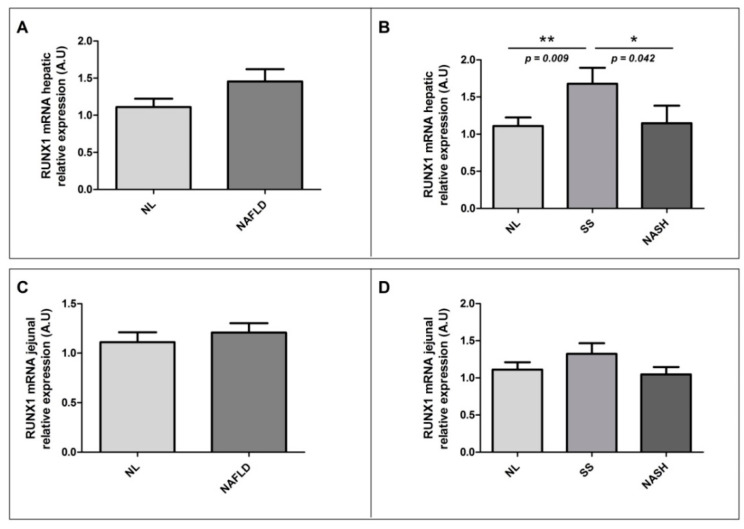
Differential relative mRNA abundance of RUNX1 in liver between (**A**) women with MO with NL histology and women with MO with NAFLD; (**B**) women with MO with NL histology, women with MO with SS, and women with MO with NASH. Differential relative mRNA abundance of RUNX1 in the jejunum between (**C**) women with MO with NL histology and women with MO with NAFLD; (**D**) women with MO with NL histology, women with MO with SS, and women with MO with NASH. A.U, arbitrary units; MO, morbidly obesity; NAFLD, nonalcoholic fatty liver disease; NL, normal liver; SS, simple steatosis; NASH, nonalcoholic steatohepatitis; RUNX1, runt-related transcription factor 1. *p* < 0.05 was considered statistically significant (* means *p* < 0.05; ** means *p* < 0.01).

To assess the main objective of this study, we evaluated the relative mRNA hepatic abundance of RUNX1 in a cohort of women with MO, with or without NAFLD. First, we did not observe significant differences in the RUNX1 hepatic expression between patients with NAFLD and those with NL histology (Figure 2A); however, when we analyzed the hepatic relative mRNA abundance of RUNX1 according to different degrees of NAFLD, we found that the RUNX1 hepatic expression was significantly higher in patients with SS than those with NL or NASH. Nevertheless, the hepatic relative mRNA abundance of RUNX1 did not show significant differences between NL and NASH patients (Figure 2B).

As an additional objective, we aimed to analyze the jejunal relative mRNA abundance of RUNX1 in our cohort of patients (Figure 2C,D). Unfortunately, there were no significant differences in jejunal RUNX1 expressions according to the presence of NAFLD or the hepatic histopathological classification.

### 2.3. Evaluation of the Immunohistochemical Analysis of RUNX1 in the Liver According to Hepatic Histology

To study RUNX1 protein expression, we performed an immunohistochemical (IHC) analysis of RUNX1 in five liver samples of any group (NL, SS, and NASH). RUNX1 nuclear positivity was almost absent in parenchymal cells such as hepatocytes, and it is observed in nonparenchymal cells such as endothelial or hepatic stellate cells.

**Figure 3:**
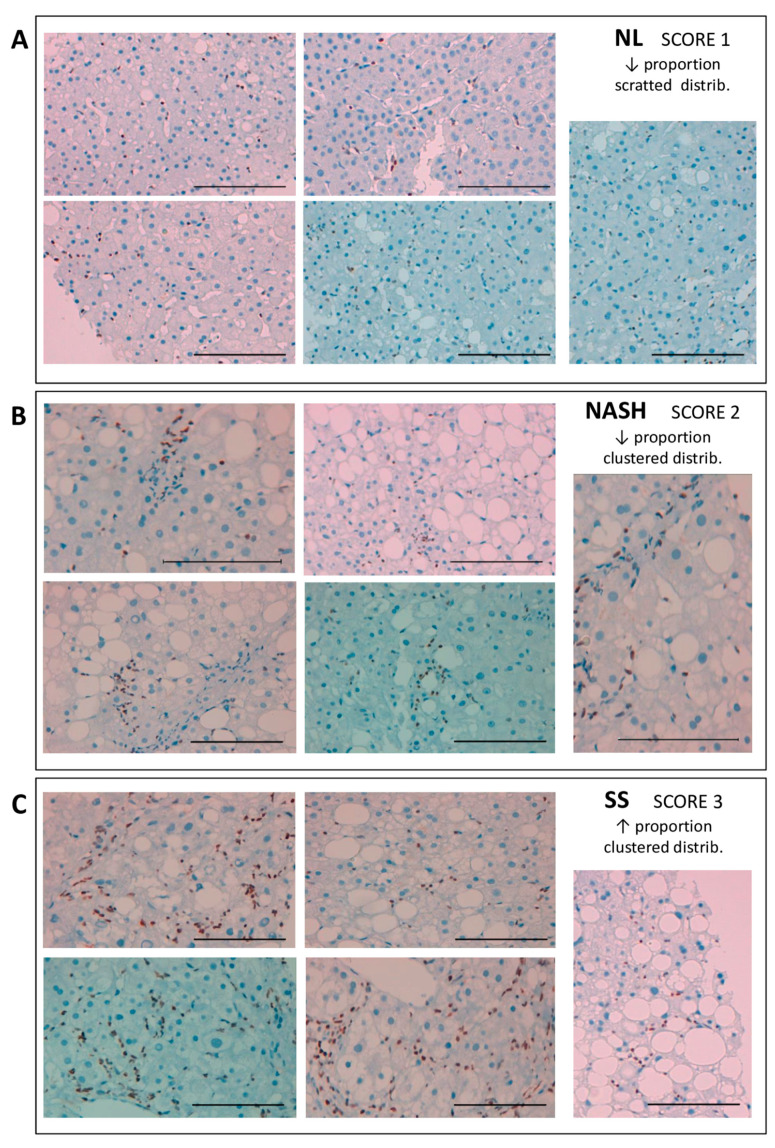
Representative images of the liver biopsies where the immunohistochemical analysis of RUNX1 was carried out. RUNX1 immunostained images (X400) in (**A**) normal liver (NL) samples, (**B**) nonalcoholic steatohepatitis (NASH) samples and (**C**) simple steatosis (SS) samples. RUNX1 positivity was mostly observed in the nonparenchymal cells. Samples were scored (from one to three) according to the RUNX1 positivity proportion (low or high) and distribution (scattered or clustered). Hematoxylin-stained nuclei were distinguishable from RUNX1-positive brown nuclei. Scale bar: 100 μm.

To perform a semi-quantitative evaluation of RUNX1 protein abundance in liver biopsies, we elaborated a score from one to three points, depending on the proportion and the distribution of the RUNX1 positivity. A score was assigned to each sample: SCORE 1 (scattered distribution and low ratio); SCORE 2 (clustered distribution and low ratio); and SCORE 3 (clustered distribution and high ratio). These are represented in Figure 3.

In this sense, we discovered that SS liver samples presented a higher proportion of RUNX1 positive cells compared to NL or NASH samples. Moreover, in SS and NASH hepatic samples, we observed a clustered distribution of the RUNX1 positive cells, while the distribution in NL was scattered. Consequently, the higher abundance of RUNX1 mRNA in SS samples was also confirmed at the protein level.

### 2.4. Evaluation of Relative mRNA Abundance of Some Lipid Metabolism-Related Genes and TLRs in the Liver and Jejunum According to Hepatic Histology

**Figure 4:**
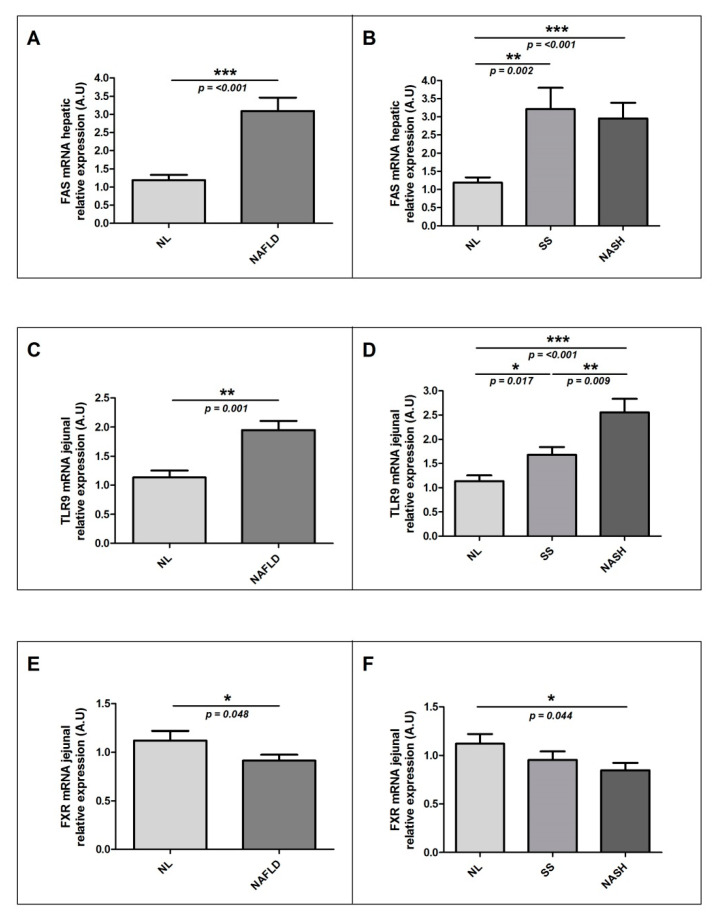
Differential relative mRNA levels of FAS in liver between (**A**) women with MO with NL histology and women with MO with NAFLD; (**B**) women with MO with NL histology, women with MO with SS, and women with MO with NASH. Differential relative mRNA levels of TLR9 in the jejunum between (**C**) women with MO with NL histology and women with MO with NAFLD; (**D**) women with MO with NL histology, women with MO with SS, and women with MO with NASH. Differential relative mRNA levels of FXR in the jejunum between (**E**) women with MO with NL histology and women with MO with NAFLD; (**F**) women with MO with NL histology, women with MO with SS, and women with MO with NASH. A.U, arbitrary units; MO, morbidly obesity; NAFLD, nonalcoholic fatty liver disease; NL, normal liver; SS, simple steatosis; NASH, nonalcoholic steatohepatitis; FAS, fatty acid synthase; TLR, toll-like receptor; FXR, farnesoid X receptor. *p* < 0.05 was considered statistically significant (* means *p* < 0.05; ** means *p* < 0.01; *** means *p* < 0.001).

Given that the expression of lipid metabolism genes was related to the expression of genes involved in the angiogenesis in adipose tissue [24], we aimed to determine the possible relationship of the RUNX1 expression with the main liver lipid metabolism-related genes (sterol regulatory element binding protein-1c (SREBP1c), fatty acid synthase (FAS), liver X receptor (LXRα), farnesoid X receptor (FXR), PPARα and PPARγ), and the TLR (TLR2, TLR4, TLR5 and TLR9) expressions in our cohort of women with MO and different hepatic histology. We observed increased levels of hepatic relative mRNA abundance of FAS in NAFLD patients as compared to those without NAFLD (Figure 4A). Subsequently, when we subclassified the patients according to hepatic histopathological classification, we found higher levels of hepatic mRNA relative abundance of FAS in SS subjects than in NL; moreover, these levels were higher in NASH than in NL patients (Figure 4B).

On other hand, when we analyzed the expression of these genes in the jejunum, we found significant differences in the relative mRNA abundance of TLR9 and FXR. In this regard, NAFLD patients showed higher levels of TLR9 and lower levels of FXR mRNA in the jejunum than those with NL histology (Figure 4C,E). When we subclassified the patients according to the hepatic histopathological classification, we then observed increased mRNA jejunal levels of TLR9 in SS as compared to those found in NL; increased levels were likewise present in NASH as compared to those in SS or NL (Figure 4D). Moreover, we could also see decreased mRNA jejunal levels of FXR in NASH patients as compared to those in NL subjects (Figure 4F).

### 2.5. Correlations of RUNX1 Relative Hepatic Expression with Different NAFLD-Related Parameters

To deepen our knowledge of RUNX1 implications in the NAFLD pathogenesis, we endeavored to analyze correlations between the RUNX1 relative expression in liver and different parameters related to NAFLD, such as weight, BMI, glucose, insulin, liver transaminases, different adipocytokines, some lipid metabolism–related genes (SREBP1c, FAS, LXRα, FXR, PPARα and PPARγ), and TLRs (TLR2, TLR4, TLR5 and TLR9). On the one hand, we found a positive correlation with the hepatic FAS expression (rho = 0.400; *p* = 0.021). On the other hand, a negative correlation with circulating resistin levels (rho = −0.375; *p* = 0.009) was found.

However, we could not find any significant correlation with inflammation or ballooning (portal inflammation *p* = 0.904, rho = −0.018; lobular inflammation *p* = 0.319, rho = −0.145; ballooning *p* = 0.363, rho = −0.134).

### 2.6. Correlations of the RUNX1 Relative Jejunal Expression with Different NAFLD-Related Parameters

When we analyzed correlations between the RUNX1 relative expression in the jejunum and other parameters, we found correlations with weight (rho = 0.266; *p* = 0.035) and fasting glucose levels (rho = −0.255; *p* = 0.044). On the other hand, we found significant correlations between the RUNX1 relative jejunal expression and PPARγ, TLR4, and TLR5 in the jejunum, as shown in Table 2.

## 3. Discussion

The novelty of the present study lies in the analysis of the hepatic and jejunal mRNA abundance of RUNX1, a molecule related to angiogenesis, in a well-characterized cohort of women with MO and NAFLD. Although there is evidence to suggest the potential role of the RUNX1 signaling pathway in NAFLD, the involvement of RUNX1 in the pathogenesis of the disease remains unknown. Therefore, we intended to deepen our knowledge about the implications of the RUNX1 expression in liver steatosis and related inflammation.

In this regard, our study reported the relative upregulation of the RUNX1 expression in the liver of patients with SS compared to that of patients with NL histology; in addition, we found downregulated RUNX1 expressions in patients with NASH compared to those in patients with SS, which was at a similar level to that found in NL patients. We were unable to find significant differences in the relative RUNX1 expression in the liver between patients with or without NAFLD, probably because the peak RUNX1 expression is found in patients with SS, and not in patients with NL or NASH. These results suggest that hepatic RUNX1 expression increases in the first steps of NAFLD, but in advanced stages of the disease, RUNX1 levels decrease, as if persistent inflammation induces the inhibition of the signaling pathway. With regard to the biological implication of RUNX1 in NAFLD progression, our IHC results supported the mRNA expression data that we obtained.

Regarding steatosis, our results are in agreement with those reported by Kaur et al. [16], suggesting the proangiogenic role of RUNX1 in repairing liver damage. In this regard, RUNX1 is notably a transcription factor that plays an important role in tissue homeostasis [25] and angiogenesis [16]. During steatosis, hepatocytes are damaged, leading to a deregulation of microvascular blood flow and lipotoxicity due to oxidative stress. These outcomes activate cytokine release, promoting a pro-inflammatory state. Initial inflammation triggers angiogenesis through different pathways, one of which is the activation of RUNX1, a pro-angiogenic factor [17].

The role of RUNX1 in NASH is unclear, and there are opposing views on the possibilities. On the one hand, Kaur et al. explained that RUNX1 expression was significantly correlated with inflammation, fibrosis, and NASH activity score in NASH patients [16], which contrasts with the results in our cohort. Perhaps the different characteristics of both cohorts can explain the differences in the results: the first cohort included patients of both sexes with NAFLD who were older and only overweight; in contrast, our cohort included only women who were younger and had severe obesity. The same authors also reported that RUNX1 acts as a pro-angiogenic factor along with other pro-angiogenic genes, such as vascular endothelial growth factor, in SS and NASH [16]. On the other hand, Liu et al. showed low levels of RUNX1 in HCC. In this respect, these authors believed that hepatic RUNX1 has a tumor suppressor role inhibiting tissue angiogenesis [11]. Our results provide new evidence on the role of RUNX in NASH in a well-characterized cohort of women with MO. In our study, the patients with NASH had decreased levels of RUNX1 mRNA abundance in the liver. To explain this finding, we hypothesized that in the advanced stages of the disease such as NASH, the tissue no longer undergoes repair. However, more studies are needed to clarify the role of RUNX1 in NASH.

In adipose tissue, the expression of genes involved in angiogenesis has a relationship with lipid metabolism gene expression [24]. Therefore, we aimed to determine the possible relationship of the RUNX1 expression with the expression of main liver lipid metabolism-related genes. First, our results showed that the mRNA of hepatic FAS, an important lipogenic enzyme, is overexpressed in subjects with NAFLD compared to those with NL. However, when we subclassified our cohort according to the hepatic histopathological classification, we found a significantly enhanced hepatic mRNA abundance of FAS in patients with SS and NASH compared to subjects with NL. In a previous study, we demonstrated that FAS is overexpressed in the liver of patients with MO and NAFLD, including SS and NASH [25]. In regard to study correlations, we found a relationship between hepatic RUNX1 expression and liver FAS expression in patients with NAFLD, which had not been previously described. These results support the assumption that RUNX1 signaling can act in parallel to hepatic lipid metabolism in these patients.

As RUNX1 is a positive regulator of the TLR4 pathway [21], and TLRs have previously been related to NAFLD [22], we studied the TLR hepatic expression in our cohort. The pathogenesis of NAFLD was previously associated with TLRs in relation to the intestinal dysbiosis that developed in these patients [26,27,28]. Intestinal dysbiosis caused by harmful alimentary habits gives rise to bacterial-derived component release; these endotoxins activate hepatic TLRs through Kupffer cells and are circulated through the entero-hepatic system, producing an inflammatory response that induces lipid accumulation in hepatocytes and promotes liver fibrosis [26].

In the present study, it is worth noting that hepatic RUNX1 and FAS expression were increased in the steatosis process of patients with MO, but in NASH, their expression was diminished or not increased. It seems that in this type of obesity, these pathways are inhibited in advanced stages of the disease, perhaps as a protective mechanism. However, the precise mechanism by which RUNX1 is deregulated in the late stages of NAFLD remains uncertain.

To identify more connections between the NAFLD pathogenesis and RUNX1, and to better understand the gut–liver axis implications [23], we also studied the jejunal expression of RUNX1, certain genes involved in lipid metabolism, and TLRs.

First, we did not find significant differences in the RUNX1 jejunal expression according to the presence of NAFLD or according to the hepatic histopathological classification; however, when we analyzed the expression of main lipid metabolism-related genes and TLR in jejunal tissue, we observed that jejunal FXR expression decreases in NAFLD and in NASH. FXR modulates different genes related to glucose homeostasis, metabolism of lipids and bile acids, and the immune response as a protective mediator [29]. Therefore, our results agree with the protective role of FXR in the gut, since jejunal FXR expression decreases as NAFLD progresses.

Moreover, as the pathogenesis of NAFLD was previously associated with TLRs in relation to the intestinal dysbiosis that developed in these patients [22,26,27,28], we studied the jejunal expression of TLR in relation to the hepatic histopathology. In this sense, we found that TLR9 jejunal expression increased as NAFLD became more severe.

We found a positive correlation between the RUNX1 jejunal expression and PPARγ jejunal mRNA abundance. In this sense, PPARγ was described as exerting anti-angiogenic [30,31] or pro-angiogenic effects [32,33]. Further studies are needed to confirm these findings. We also observed certain interesting correlations between jejunal RUNX1 expression and jejunal TLR expression—a positive correlation with TLR4 and TLR5 was found. Additionally, the increased intestinal expression of TLRs has been described in different bowel diseases [16,34] and even in NAFLD [8], suggesting that the innate immune system may play an important role in the pathophysiology of NAFLD [35]. However, in the present study, we did not find significant differences in jejunal TLR expression in NAFLD; therefore, it is complicated to explain this correlation.

**Figure 5:**
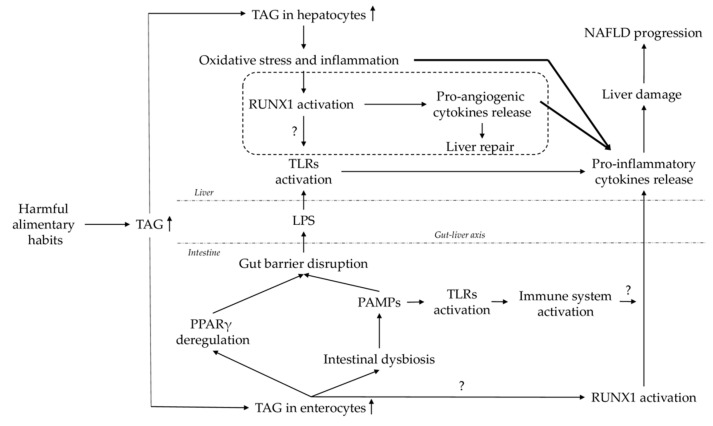
Role of RUNX1 in gut–liver axis implicated in NAFLD pathogenesis. TAG, triglycerides; RUNX1, runt-related transcription factor 1; TLRs, toll-like receptors; NAFLD, nonalcoholic fatty liver disease; LPS, lipopolysaccharides; PPARγ, peroxisome proliferator-activated receptor gamma; PAMPs, pathogen-associated molecular patterns.

In summary, our findings indicate that in women with MO, the RUNX1 signaling pathway in the liver seems to be upregulated in SS and downregulated in NASH. The relationship with hepatic FAS expression suggests that certain mechanisms, such as hepatic lipogenic or pro-angiogenic pathways, seem to be attenuated in advanced stages of NAFLD. In regard to the role of RUNX1 signaling in the jejunum, we found an important association with PPARγ and TLRs, which corroborates the key role of the gut–liver axis in the development of NAFLD. These main findings and hypotheses are represented in the diagram below (Figure 5).

Our cohort of women with MO made it possible to establish some relationships between RUNX1 and NAFLD without the interference of confounding factors such as sex or age. However, these results cannot be extrapolated to other groups with obesity, people of normal weight, or people with overweight. Further studies, including those of these cohorts, would be useful to validate our findings.

## 4. Materials and Methods

### 4.1. Subjects

The study was approved by the institutional review board (Institut Investigació Sanitària Pere Virgili (IISPV) CEIm; 23c/2015; 11 May 2015), and all participants gave written informed consent. The study population consisted of 69 Caucasian women with MO (BMI > 40 kg/m^2^). Liver and jejunum biopsies were obtained during a planned laparoscopic bariatric surgery. All liver biopsies were indicated for clinical diagnosis. The exclusion criteria were as follows: (1) subjects who had alcohol consumption higher than 10 g/day; (2) patients who had acute or chronic hepatic, inflammatory, infectious, or neoplasic diseases; (3) women who were menopausal or undergoing contraceptive treatment; (4) women with diabetes receiving pioglitazone or insulin; and (5) patients treated with antibiotics in the previous 4 weeks.

### 4.2. Sample Size

Accepting an α risk of 0.05 and a β risk of less than 0.2 in a bilateral contrast, 24 subjects per group were needed to detect a difference of ≥0.2 units. It was assumed that the common standard deviation would be 0.3.

### 4.3. Liver Pathology

**Figure 6:**
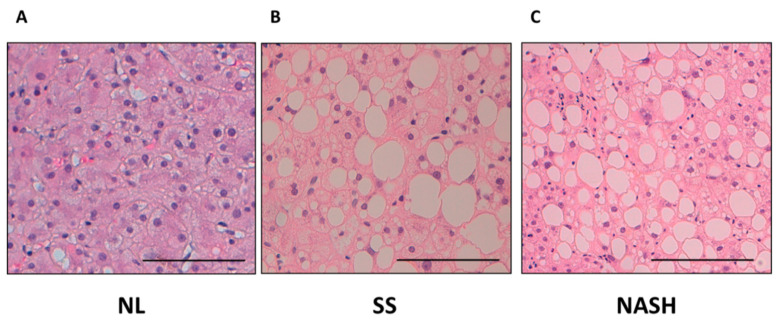
Histological images of liver biopsies stained with eosin-hematoxylin from (**A**) women with MO with NL histology (**B**) women with MO with SS, and (**C**) women with MO with NASH. X400, scale bar: 100 μm.

Liver samples were stained with hematoxylin and eosin, and Manson’s trichrome stains, and scored by experienced hepatopathologists using other methods previously described [5]. According to their liver pathology, women with MO were classified into NL histology (*n* = 28) and NAFLD (*n* = 41). Patients were then subclassified into NL histology (*n* = 28), SS (micro/macrovesicular steatosis without inflammation or fibrosis, *n* = 24), and NASH (Brunt grades 1–2, *n* = 17). None of the patients with NASH in our cohort presented fibrosis. In order to give visual information, Figure 6 shows the histological features, grading, and staging of NL and NAFLD (SS and NASH) with our own images.

### 4.4. Biochemical Analyses

All of the subjects underwent physical, anthropometric, and biochemical assessments. Blood extraction was performed by specialized nurses through a BD Vacutainer^®^ system, after overnight fasting and before bariatric surgery. Venous blood samples were obtained in ethylenediaminetetraacetic acid tubes, which were separated in plasma and serum aliquots by centrifugation (3500 rpm, 4 °C, 15 min). Biochemical parameters were analyzed using a conventional automated analyzer. Insulin resistance was estimated using HOMA1-IR.

Cytokines, such as IL-1β, IL-6, IL-8, IL-10, TNF-α, adiponectin, and resistin were determined using multiplex sandwich immunoassays and the MILLIPLEX MAP Human Adipokine Magnetic Bead Panel 1 (HADK1MAG-61K, Millipore, Billerica, MA, USA), the MILLIPLEX MAP Human High-Sensitivity T Cell Panel (HSTCMAG28SK, Millipore, Billerica, MA, USA), and the Bio-Plex 200 instrument at the Center for Omic Sciences (Universitat Rovira i Virgili), according to the manufacturer’s instructions.

### 4.5. Gene Expression in the Liver and Jejunum

Liver and jejunum samples collected during bariatric surgery were conserved in RNAlater (Qiagen, Hilden, Germany) at 4 °C and then processed and stored at −80 °C. Total RNA was extracted from both tissues by using the RNeasy mini kit (Qiagen, Barcelona, Spain). Reverse transcription to cDNA was performed with the High-Capacity RNA-to-cDNA Kit (Applied Biosystems, Madrid, Spain). Real-time quantitative PCR was performed with the TaqMan Assay predesigned by Applied Biosystems for the detection of RUNX1 (Hs01021970_m1), TLR2 (Hs02621280_s1), TLR4 (Hs00152939_m1), TLR5 (Hs05021301_s1), TLR9 (Hs00370913_s1), SREBP1c (Hs01088691_m1), LXRα (Hs00173195_m1), FAS (Hs00188012_m1), FXR (Hs01026590_m1), PPARα (Hs00947538_m1), and PPARγ (Hs01115513_m1). The expression of each gene was calculated relative to the expression of 18S RNA (Fn04646250_s1) for liver genes, and glyceraldehyde-3-phosphate dehydrogenase (GAPDH) (Hs02786624_g1) for genes in the jejunum, after which it was normalized using the control group (NL) as a reference. All reactions were duplicated in 96-well plates using the 7900HT Fast Real-Time PCR system (Applied Biosystem, Foster City, CA, USA).

### 4.6. Immunohistochemistry Analysis

Samples of human liver tissues (5 samples from any group: NL, SS, and NASH) were fixed and stained according to Kaur et al. IHC analysis [16]. Details of the IHC analysis were: RUNX1 primary antibody (Cat. Num: sc-365644; host: mouse; fluorochrome: unconjugated; dilution: 1/100) provided by Santa Cruz Biotechnology Inc., Dallas, TX, USA. RUNX1 positive cells across the whole tissue sample were evaluated under a microscope (X400) by a specialist hepatopathologist.

The immunoreactive index was scored depending on the proportion of RUNX1 positivity (RUNX1 positive nonparenchymal cells: total of parenchymal and nonparenchymal cells ratio) and the distribution of RUNX1 positive cells, as shown in Table 3.

The evaluation of these parameters was not carried out in a representative field since proportion and distribution of the RUNX1 positivity were not found homogeneously throughout the tissue sample; a general evaluation of the tissue is needed to be more precise in the score assignment, in the same way that the histopathological diagnosis was made.

### 4.7. Statistical Analysis

The data was analyzed using the SPSS/PC+ for Windows statistical package (version 23.0; SPSS, Chicago, IL, USA). The Kolmogorov–Smirnov test was used to assess the distribution of variables. Continuous variables were reported as the mean (SD); non-continuous variables were reported as the median and the interquartile range. The different comparative analyses were performed using a nonparametric Mann–Whitney *U* test or Kruskal–Wallis test, according to the presence of two or more groups. The strength of the association between variables was calculated using Spearman’s method. *p*-values <0.05 were statistically significant.

## 5. Conclusions

The hepatic expression of RUNX1 appears to be involved in the first steps of NAFLD, perhaps with a pro-angiogenic role in order to repair hepatic damage. However, RUNX1 seems to be downregulated in NASH. These facts suggest that RUNX1 may have a protective role in NAFLD progression in patients with morbid obesity.

## Figures and Tables

**Table 1:** Anthropometric and biochemical variables of the cohort with MO, classified according to the hepatic histopathological characteristics.

Variables	NL	NAFLD	SS	NASH
	(*n* = 28)	(*n* = 41)	(*n* = 24)	(*n* = 17)
Weight (kg)	116.50 (107.25–130.50)	112.40 (106.00–128.00)	113.20 (108.33–128.00)	112.00 (104.65–125.00)
BMI (kg/m^2^)	43.30 (40.94–46.47)	44.46 (40.84–46.60)	44.35 (40.82–46.83)	44.46 (40.76–46.03)
Glucose (mg/dL)	85.50 (76.25–93.00)	93.00 (84.00–105.00) *	93.50 (85.75–107.00) ^#^	91.00 (82.50–101.20)
Insulin (mUI/L)	9.43 (5.59–16.21)	9.63 (5.88–14.52)	11.27 (7.81–14.51)	6.57 (5.09–23.04)
HOMA1-IR	2.07 (1.06–3.35)	2.14 (1.27–3.97)	2.70 (1.59–4.77)	1.57 (1.05–4.12)
HbA1c (%)	5.40 (5.30–5.70)	5.60 (5.20–6.05)	5.60 (5.25–6.03)	5.50 (5.20–6.10)
Cholesterol (mg/dL)	171.88 ± 36.20	179.07 ± 38.80	174.42 ± 35.41	185.28 ± 43.39
HDL-C (mg/dL)	40.84 ± 9.89	41.04 ± 10.95	42.56 ± 12.38	38.89 ± 8.47
LDL-C (mg/dL)	108.16 ± 27.94	104.48 ± 30.86	104.39 ± 31.21	104.62 ± 31.58
Triglycerides (mg/dL)	106.00 (89.00–136.00)	132.00 (91.00–189.00)	129.50 (85.75–175.50)	140.00 (106.00–247.00)
AST (UI/L)	20.50 (15.75–36.25)	23.50 (17.00–41.75)	23.00 (17.00–35.00)	24.00 (17.00–43.00)
ALT (UI/L)	22.00 (16.00–27.00)	31.00 (21.00–37.00)	31.00 (23.00–35.75) ^#^	30.00 (15.50–40.00)
GGT (UI/L)	18.00 (16.00–27.00)	22.00 (16.00–27.00)	21.00 (16.25–30.50)	25.00 (15.00–27.00)
ALP (Ul/L)	60.42 ± 13.09	70.67 ± 13.01 *	75.80 ± 11.66 ^#^	62.77 ± 11.16 ^&^
LDH (Ul/L)	378.65 ± 54.30	421.00 ± 82.47	428.08 ± 79.38	411.80 ± 89.76
SBP (mmHg)	119.00 ± 18.26	117.29 ± 13.86	120.09 ± 13.41	113.44 ± 13.96
DBP (mmHg)	63.00 (57.75–75.75)	62.00 (59.00–71.25)	62.00 (59.00–72.50)	65.50 (56.75–70.75)
IL-1β (pg/mL)	4.14 ± 2.07	5.10 ± 2.93	4.91 ± 2.15	5.38 ± 3.84
IL-6 (pg/mL)	4.24 ± 2.51	3.65 ± 1.83	3.40 ± 1.54	4.01 ± 2.20
IL-8 (pg/mL)	2.84 (2.30–3.48) ^¬^	3.61 (2.82–4.54) *	3.47 (2.56–4.09)	4.18 (3.12–5.29) ^&^
TNF-α (pg/mL)	10.07 (7.92–11.71)	10.44 (7.50–12.26)	9.73 (7.36–11.23)	11.31 (7.80–12.57)
IL-10 (pg/mL)	3.99 (1.44–7.30)	2.92 (1.49–5.95)	2.79 (1.78–9.96)	2.93 (1.16–5.87)
Adiponectin (ng/mL)	14,209.54 ± 6519.51	12,187.96 ± 6707.07	13,263.39 ± 7238.02	10,709.23 ± 5795.64
Resistin (ng/mL)	26.16 (19.72–34.94)	26.27 (19.03–32.84)	26.27 (20.95–32.84)	27.10 (18.56–43.83)

NL, normal liver; SS, simple steatosis; NAFLD, nonalcoholic fatty liver disease; NASH, nonalcoholic steatohepatitis; BMI, body mass index; HOMA1-IR, homeostatic model assessment method 1 of insulin resistance; HbA1c, glycosylated hemoglobin; HDL-C, high density lipoprotein cholesterol; LDL-C, low density lipoprotein cholesterol; AST, aspartate aminotransferase; ALT, alanine aminotransferase; GGT, gamma-glutamyltransferase; ALP, alkaline phosphatase; LDH, lactate dehydrogenase; SBP, systolic blood pressure; DBP, diastolic blood pressure; IL, interleukin; TNF-α, tumor necrosis factor α. Data are expressed as the mean ± standard deviation or median (interquartile range), depending on the distribution of the variables. * Significant differences between NL group and NAFLD group (*p* < 0.05). ^#^ Significant differences between NL group and SS group (*p* < 0.05). ^&^ Significant differences between SS group and NASH group (*p* < 0.05). ^¬^ Significant differences between NL group and NASH group (*p* < 0.05).

**Table 2:** Significant correlations between the RUNX1 relative jejunal expression and other genes in the jejunum.

Genes	RUNX1 mRNA Jejunal R.E.	
	rho	*p* Value
TLR4 mRNA jejunal R.E.	0.421	0.001
TLR5 mRNA jejunal R.E.	0.302	0.031
PPARγ mRNA jejunal R.E.	0.352	0.008

TLR, toll-like receptor; PPARγ, peroxisome proliferator-activated receptor gamma; RUNX1, runt-related transcription factor 1; R.E., relative expression. Data are expressed as the correlation coefficient rho of Spearman and *p*-value (*p* < 0.05 was considered statistically significant).

**Table 3:** Description of the immunoreactive score in liver samples depending on the proportion and the distribution of the RUNX1 positive cells.

SCORE	Proportion	Distribution
1	↓	scattered
2	↓	clustered
3	↑	clustered

Proportion is based on ↓ (low) or ↑ (high) positive nonparenchymal cells: total cells; distribution is based on scattered positive cells or clustered positive cells.

## Data Availability

Not applicable.

## Abbreviations

ALP	Alkaline phosphatase
ALT	Alanine aminotransferase
AST	Aspartate aminotransferase
BMI	Body mass index
DBP	Diastolic blood pressure
FAS	Fatty acid synthase
FXR	Farnesoid X receptor
GGT	Gamma-glutamyltransferase
HbA1c	Glycosylated hemoglobin
HCC	Hepatocellular carcinoma
HDL-C	High density lipoprotein cholesterol
HOMA1-IR	Homeostatic model assessment method 1 of insulin resistance
IL	Interleukin
LDH	Lactate dehydrogenase
LDL-C	Low density lipoprotein cholesterol
LXR	Liver X receptor
MetS	Metabolic syndrome
MO	Morbid obesity
NAFLD	Nonalcoholic fatty liver disease
NASH	Nonalcoholic steatohepatitis
NL	Normal liver
PPARγ	Peroxisome proliferator-activated receptor γ
RUNX1	Runt-related transcription factor 1
SBP	Systolic blood pressure
SREBP1c	Sterol regulatory element binding protein-1c
SS	Simple steatosis
TNF-α	Tumor necrosis factor α
TLR	Toll-like receptor
